# Supplementary material for: Novel symmetrical bifacial flexible CZTSSe thin film solar cells for indoor photovoltaic applications
Source: Nat Commun. 2021 May 25;12:3107. doi: 10.1038/s41467-021-23343-1 (PMC8149396; doi:10.1038/s41467-021-23343-1)
Supplement: Supplementary file 1 — Supplementary Information [file 41467_2021_23343_MOESM1_ESM.pdf]

Supplementary Information

Novel Symmetrical Bifacial Flexible CZTSSe Thin Film Solar Cells for Indoor Photovoltaic Applications

Hui Deng[1], Quanzhen Sun[1], Zhiyuan Yang[1], Wangyang Li[1], Qiong Yan[1], Caixia Zhang[1,2], Qiao Zheng[1,2], Xinghui Wang[1], Yunfeng Lai[1,2], Shuying Cheng[1,2,*]

[1] College of Physics and Information Engineering, Institute of Micro-Nano Devices and Solar Cells, Fuzhou University, Fuzhou, 350108, P. R. China.

[2]Jiangsu Collaborative Innovation Center of Photovoltaic Science and Engineering, Changzhou, 213164, P. R. China.

* Corresponding author E-mail: sycheng@fzu.edu.cn (S. Cheng).

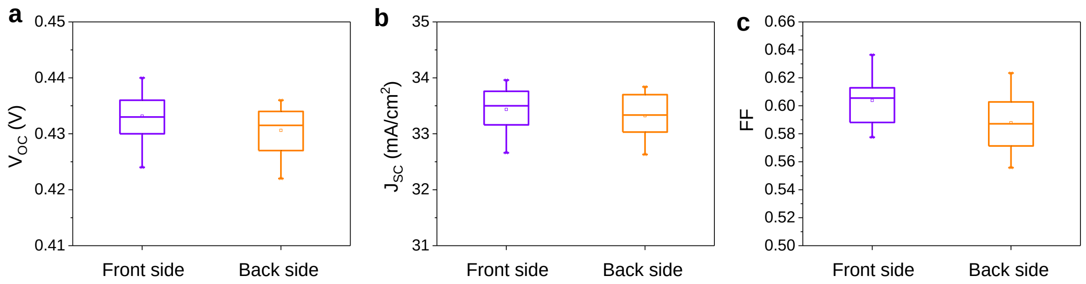

Supplementary Figure 1 Statistical distribution boxplots of bifacial solar cells. **a** the V_{OC} distribution boxplots. **b** the J_{SC} distribution boxplots. **c** the FF distribution boxplots.

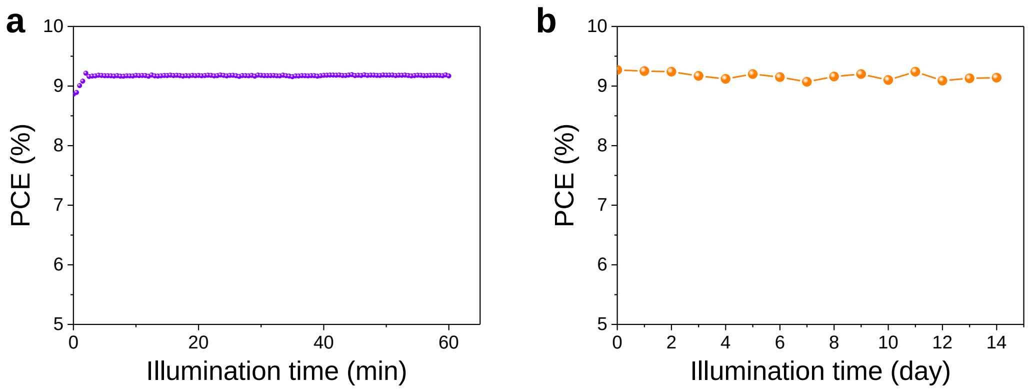

Supplementary Figure 2 Stability testing of the flexible bifacial CZTSSe solar cells. **a** PCE stability testing by continuous illumination for 1 hour. **b** PCE evolutions by continuous illumination for several days. The solar cell device was tested once a day.

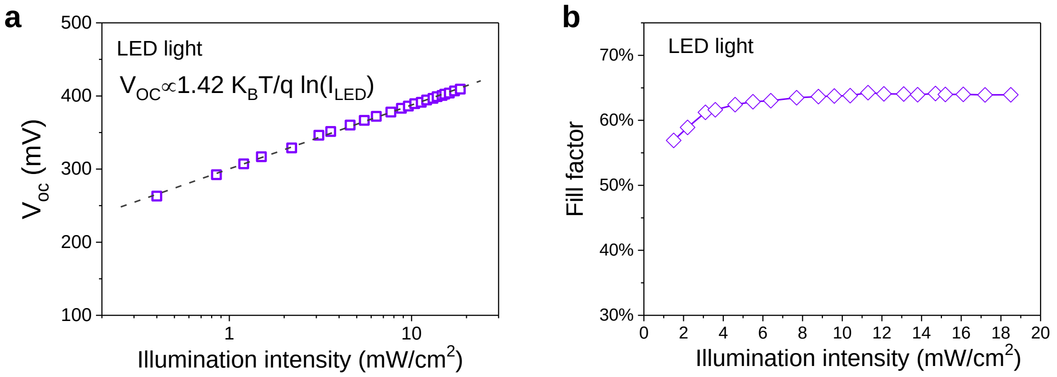

Supplementary Figure 3. a The V_{OC} and **b** Fill factor evolution with LED light intensity.

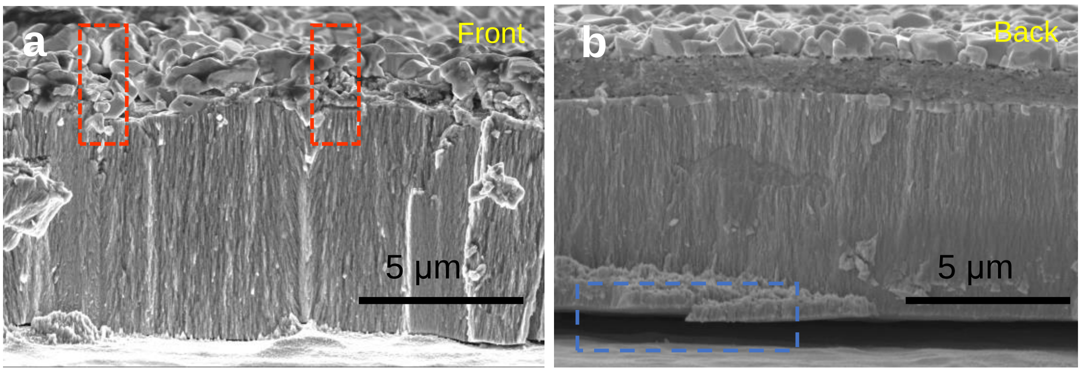

Supplementary Figure 4 Cross-section SEM image of the flexible bifacial CZTSSe solar cells after bending. Cross-section SEM image of **a** the front cell and **b** the back cell after bending 4000 times at the fixed angle of 70° (numerous bending times and large bending angle).

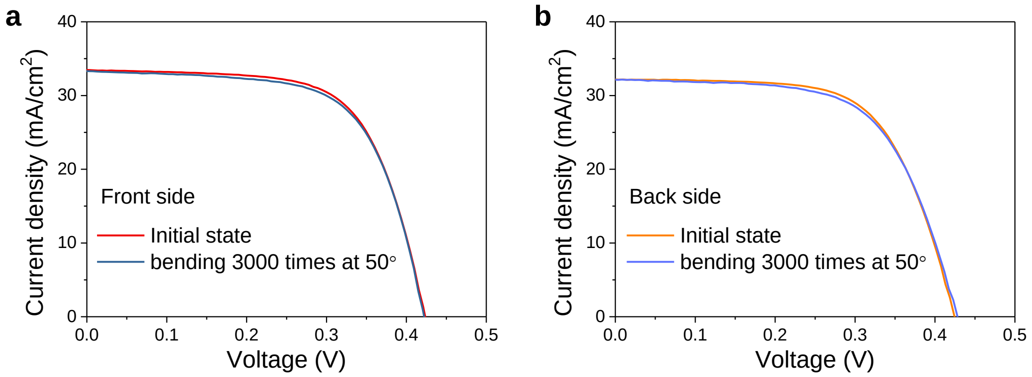

Supplementary Figure 5 J-V curves of **a** the front cell and **b** the back cell after bending 3000 times at the fixed angle of 50°.

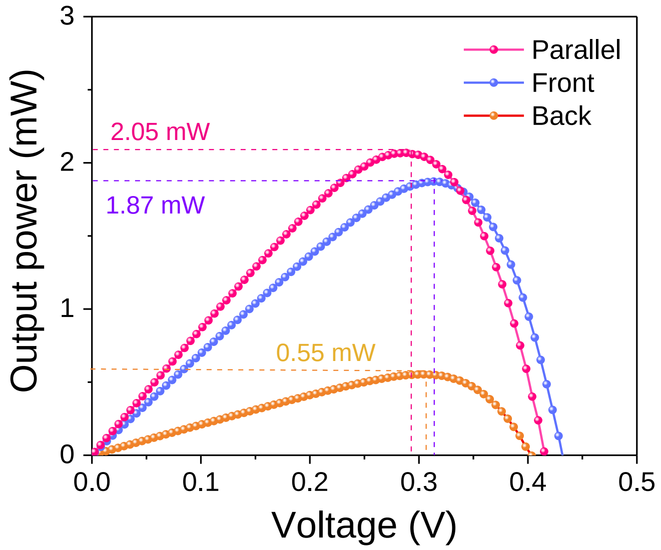

Supplementary Figure 6 The output power-voltage (P-V) relationship of double-sided cells in parallel. The front cell is illuminated by 1 sun and the back side is illuminated by 0.3 sun.
