# Supplementary material for: Novel symmetrical bifacial flexible CZTSSe thin film solar cells for indoor photovoltaic applications
Source: Nat Commun. 2021 May 25;12:3107. doi: 10.1038/s41467-021-23343-1 (PMC8149396; doi:10.1038/s41467-021-23343-1)
Supplement: Supplementary file 3 — Solar Cells Reporting Summary [file 41467_2021_23343_MOESM3_ESM.pdf]

nature research

Corresponding author(s): Shuying Cheng

Solar Cells Reporting Summary

Nature Research wishes to improve the reproducibility of the work that we publish. This form is intended for publication with all accepted papers reporting the characterization of photovoltaic devices and provides structure for consistency and transparency in reporting. Some list items might not apply to an individual manuscript, but all fields must be completed for clarity.

For further information on Nature Research policies, including our data availability policy, see Authors & Referees.

Experimental design

Please check: are the following details reported in the manuscript?

1. Dimensions

Item	Yes	No	Response
Area of the tested solar cells	☒	☐	Area of the tested solar cells are described in the section of Methods.
Method used to determine the device area	☒	☐	Every device is divided by blade and its area is 0.21 cm2 except grid area. The related information is shown in the section of Methods.

2. Current-voltage characterization

Item	Yes	No	Response
Current density-voltage (J-V) plots in both forward and backward direction	☐	☒	There is no reported influence of scan direction on CZTS-based thin-film solar cell J-V characteristics.
Voltage scan conditions *For instance: scan direction, speed, dwell times*	☒	☐	The related information is shown in the section of Methods.
Test environment *For instance: characterization temperature, in air or in glove box*	☒	☐	The related information is described in in the section of Methods.
Protocol for preconditioning of the device before its characterization	☒	☐	The related information is described in the section of Methods.
Stability of the J-V characteristic *Verified with time evolution of the maximum power point or with the photocurrent at maximum power point; see ref. 7 for details.*	☒	☐	The related information is shown in Supplementary Fig. 2.

3. Hysteresis or any other unusual behaviour

Item	Yes	No	Response
Description of the unusual behaviour observed during the characterization	☐	☒	We did not found hysteresis or unusual behaviour during the characterization.
Related experimental data	☐	☒	Experimental data is shown in the section of Methods and Discussion. We did not found unusual behaviour during the characterization.

4. Efficiency

Item	Yes	No	Response
External quantum efficiency (EQE) or incident photons to current efficiency (IPCE)	☒	☐	The related EQE information is shown in Fig 4c.
A comparison between the integrated response under the standard reference spectrum and the response measure under the simulator	☒	☐	The related information is described in the section of Methods.
For tandem solar cells, the bias illumination and bias voltage used for each subcell	☐	☒	This work doesn't involve tandem solar cells.

5. Calibration

Item	Yes	No	Response
Light source and reference cell or sensor used for the characterization	☒	☐	The related information is described in the section of Methods.
Confirmation that the reference cell was calibrated and certified	☒	☐	The related information is described in the section of Methods.

Calculation of spectral mismatch between the reference cell and the devices under test

- [ ] Yes
- [x] No

We tested the devices by standard solar simulator. Spectra are calibrated by a standard silicon solar cell.

6. Mask/aperture

Size of the mask/aperture used during testing

- [ ] Yes
- [x] No

Each device is independent and separated.

Variation of the measured short-circuit current density with the mask/aperture area

- [ ] Yes
- [x] No

Each device is independent and separated,thus we did not limit light by mask.

7. Performance certification

Identity of the independent certification laboratory that confirmed the photovoltaic performance

- [ ] Yes
- [x] No

We don't make efficiency certificate.

A copy of any certificate(s)
Provide in Supplementary Information

- [ ] Yes
- [x] No

We don't make efficiency certificate.

8. Statistics

Number of solar cells tested

- [x] Yes
- [ ] No

The statistical analysis is from 18 devices in each group. The related information is shown in the section of Methods and Results.

Statistical analysis of the device performance

- [x] Yes
- [ ] No

The related information is shown in Fig. 4a and Supplementary Fig. 1.

9. Long-term stability analysis

Type of analysis, bias conditions and environmental conditions
For instance: illumination type, temperature, atmosphere humidity, encapsulation method, preconditioning temperature

- [x] Yes
- [ ] No

The related information is shown in Supplementary Fig. 2.
